# TransBridge: A Transparent Communication Middleware with Unified RoCE and TCP Semantics

**DOI:** 10.3390/s26082482

**Published:** 2026-04-17

**Authors:** Cong Zhou, Yulei Yuan, Peng Xun

**Affiliations:** College of Computer Science and Technology, National University of Defense Technology, Changsha 410000, China; zhoucong19a@nudt.edu.cn (C.Z.); xunpeng12@nudt.edu.cn (P.X.)

**Keywords:** RoCE, RDMA, socket, user-space, TCP

## Abstract

In low-latency edge-intelligence scenarios such as autonomous driving and industrial edge analytics, the processing of large-scale sensor data imposes extremely stringent requirements on communication latency. However, the high overhead of the traditional TCP protocol makes it difficult to satisfy such demands, while the semantic gap between the high-performance RoCE protocol and the standard Socket API prevents existing applications from directly exploiting its advantages. To address this problem, this paper proposes TransBridge, a lightweight user-space communication middleware that transparently bridges TCP and RoCE. Its design is realized through three key innovations: a transparent user-space compatibility architecture that enables unmodified Socket-based applications to benefit from RoCE performance; a microsecond-level low-latency transmission engine that bypasses kernel and protocol stack overhead; and a lightweight lock-free resource management mechanism based on a decentralized peer-to-peer architecture and deferred buffer updates. Experiments on a real RoCE network show that TransBridge significantly outperforms mainstream schemes: it achieves an average round-trip latency of 5.926 μs for 16 B messages and a throughput of 20.254 Gbps for 16 KB messages; in the Fast DDS application-level evaluation, it achieves a throughput of 188 Mbps and an average round-trip latency of about 150 μs. The results indicate that TransBridge can provide transparent and effective RoCE acceleration for existing Socket-based applications in resource-constrained edge environments.

## 1. Introduction

Edge-intelligence applications, such as multi-sensor perception fusion in autonomous driving, remote robotic teleoperation, and real-time video analytics and data aggregation in industrial environments, typically impose stringent requirements on communication systems in terms of low latency, high responsiveness, and efficient resource utilization [1,2]. In such scenarios, data exchange among distributed nodes is often latency-critical, while the participating edge devices are usually constrained by limited CPU, memory, and energy budgets. Although the standard TCP/Socket interface provides strong generality and broad software-ecosystem compatibility, its communication path still relies on system calls, user/kernel transitions, data copying, and kernel protocol-stack processing. These software overheads increasingly become a noticeable bottleneck in resource-constrained edge environments that require real-time responsiveness [3,4,5,6,7,8,9,10,11,12,13,14,15,16,17,18,19,20]. Therefore, there is a strong need for a communication mechanism that can reduce software overhead while still supporting existing edge applications in a transparent manner.

To alleviate the above limitations of the conventional TCP/Socket path, RDMA/RoCE has been increasingly explored as a promising communication approach for latency-critical edge-intelligence scenarios. In recent years, RDMA has gradually extended beyond traditional datacenters and high-performance computing environments to domains such as autonomous driving, teleoperation, and industrial control. For example, Ref. [21] employs Software RDMA (Soft-RoCE) in a distributed autonomous-driving platform to optimize data transfer across heterogeneous computing units and further improves determinism through an integrated deterministic scheduler. Ref. [22] combines RDMA acceleration with high-precision bilateral control in a remote robotic teleoperation scenario, achieving stable low-latency force-feedback control over distances exceeding 100 km, with approximately 1 ms bidirectional control latency. These studies indicate that RDMA is not merely a high-performance interconnect for datacenters, but also a feasible communication candidate for supporting latency-critical edge-intelligence applications.

However, despite the strong potential of RDMA in the above scenarios, the vast majority of existing network applications are still built on the standard Socket/TCP programming model and therefore cannot directly exploit the high-performance data plane provided by RoCE. The key difficulty is not merely that Socket and RDMA Verbs differ in interface style, but that existing applications are already deeply coupled with the stream-oriented Socket abstraction and cannot be practically rewritten around the low-level RDMA programming model. Socket communication follows a stream-oriented abstraction, relying on dynamically managed kernel buffers and synchronous I/O readiness semantics, whereas RoCE requires explicit memory registration, queue-pair management, and asynchronous completion handling [4,5]. As a result, this gap is not limited to the API level; it also extends to connection establishment, buffer organization, event notification, and error handling, making it highly difficult for existing TCP applications to transparently migrate to RoCE-based communication paths without code modification [11,20,23,24,25].

To bridge TCP and RDMA/RoCE, a number of compatibility and acceleration approaches have already been proposed. However, most of these solutions are primarily designed for resource-rich datacenter environments, and typically rely on centralized resource coordination, relatively heavy software protocol processing, complex connection multiplexing, or hybrid kernel/user-space event-handling paths [4,5,6,7,8,9,10,11,12]. In some designs, connection management, resource requests, and event notifications further depend on separate monitoring processes or runtime components, which introduces additional inter-process communication, context-switching, and state-synchronization overhead. In server environments with sufficient resources, such overheads can often be amortized by throughput gains. On edge nodes, however, limited CPU and memory resources make the costs introduced by centralized coordination, frequent synchronization, inter-process communication, and complicated control paths much more pronounced. In other words, although existing approaches have demonstrated the feasibility of Socket–RDMA compatibility, they still do not simultaneously satisfy the key requirements of transparency, lightweight runtime behavior, and low software overhead in resource-constrained edge environments. Therefore, the key problem addressed in this paper is not merely how to combine TCP compatibility with RoCE performance, but rather: how to transparently and efficiently map standard Socket applications onto RoCE-based high-performance communication paths in resource-constrained edge environments with low software overhead.

To address this problem, this paper presents TransBridge, a lightweight user-space communication middleware that establishes a transparent bridge between the standard Socket interface and the RoCE high-performance data plane. Rather than introducing another general-purpose Socket-over-RDMA framework, this work focuses on a lightweight and transparently compatible mechanism for edge scenarios, aiming to preserve the standard Socket semantics required by existing applications while minimizing the runtime overhead of mapping them onto a RoCE-based communication path. TransBridge does not attempt to reconstruct a complete software TCP/IP stack in user space. Instead, it is built around three mutually complementary design principles. First, to avoid modifying existing applications, it achieves transparent compatibility with standard Socket APIs through a user-space interception mechanism, enabling them to access the accelerated path without code changes. Second, because edge nodes are more sensitive to software overhead, it constructs a low-latency RoCE-based data plane without relying on a conventional software protocol stack, and reduces protocol overhead while preserving TCP stream semantics through intermediate buffering, aggregated transmission, and receiver-window feedback. Third, to avoid the synchronization burden and performance bottlenecks caused by centralized coordination, it employs decentralized lock-free resource management and event handling, thereby improving runtime efficiency on resource-constrained edge nodes. Through this design, TransBridge preserves the standard Socket programming model while allowing applications to transparently benefit from RoCE acceleration.

The main contributions of this paper are summarized as follows:

- A transparent Socket–RoCE compatibility framework for edge-intelligence scenarios. The proposed framework transparently maps eligible communication paths onto the RoCE data plane through user-space API interception and redirection without requiring any modification to application code, thereby preserving the standard Socket programming interface while enabling high-performance communication.
- A low-latency transmission mechanism without rebuilding a full software protocol stack. This mechanism constructs a lightweight user-space transmission path for TCP semantics, avoids layered processing in the conventional software protocol stack on the data plane, and achieves efficient stream-oriented communication through intermediate buffering, aggregated transmission, and receiver-window feedback.
- A decentralized lock-free resource-management and unified event-handling mechanism. By avoiding centralized monitoring and re-synchronization paths, the system reduces lock contention and coordination overhead, and effectively maps low-level asynchronous RoCE completion events into application-visible Socket readiness semantics, thereby improving runtime efficiency on resource-constrained edge nodes.

We implement TransBridge on a real 100 Gbps RoCE testbed and evaluate it against multiple representative communication solutions. Experimental results show that, while preserving compatibility with the standard Socket API, TransBridge significantly reduces small-message communication latency and achieves higher throughput for medium-sized messages. These results demonstrate that TransBridge provides a practical and effective solution for transparent high-performance communication in edge-intelligence scenarios.

## 2. Technical Background and Related Work

This section first briefly introduces the RDMA communication model and the basic terminology involved in the subsequent design of this paper, then categorizes and analyzes existing Socket–RDMA integration approaches, and finally summarizes the limitations of prior work in resource-constrained edge-intelligence scenarios so as to clarify the research positioning of this work.

### 2.1. RDMA Communication Model and Background

**Figure 1:**
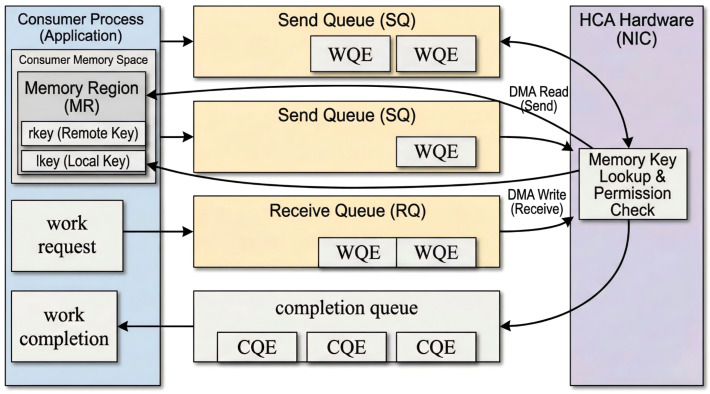
Illustration of the RDMA communication model.

Remote Direct Memory Access (RDMA) is a communication mechanism designed for high-performance networking environments, with the core goal of enabling direct data exchange between user-space memory regions across nodes while minimizing host CPU involvement. Unlike the traditional communication model based on Socket, RDMA communication mainly revolves around three core objects: the queue pair (QP), the memory region (MR), and the completion queue (CQ). Among them, the QP serves as the basic communication endpoint and consists of a send queue and a receive queue for posting send, receive, read, and write requests; the MR represents a user-space memory region that can be directly accessed by the NIC after registration; and the CQ is used to receive asynchronous completion events, where each Completion Queue Entry (CQE) reports the execution result of a request [3,4,5,6,7,26,27]. Applications drive data transfer by posting Work Requests (WRs) to QPs and describing data buffers with Scatter/Gather Entries (SGEs). The overall communication relationship is shown in Figure 1.

In actual communication, the two communicating parties usually need to exchange the metadata required to establish the data path through an independent control path, such as the Queue Pair Number (QPN), the receive-buffer address, and the Remote Key (RKey). On this basis, subsequent data-plane transmission can be further offloaded to the NIC hardware. Depending on the transmission semantics, RDMA typically provides multiple service types, among which Reliable Connection (RC) offers ordered and reliable transmission semantics, while Unreliable Datagram (UD) retains the characteristics of connectionless datagram delivery. Since the remainder of this paper will frequently involve terms such as QP, MR, CQ, QPN, RKey, and RC/UD, it is necessary to briefly introduce the RDMA communication model here.

### 2.2. Existing Socket–RDMA Integration Approaches

To address the practical challenge that traditional Socket applications cannot directly exploit the high-performance RDMA/RoCE data plane, a variety of “Socket over RDMA” compatibility and acceleration approaches have been proposed. In general, these studies can be divided into two categories: one implements cooperation between Socket and RDMA inside the operating-system kernel, while the other intercepts Socket calls in user space and directly constructs a high-performance data path based on the RDMA Verbs interface. The two categories differ in transparency, deployment model, and performance overhead [4,28,29,30].

Among kernel-space solutions, SMC-R [7] (Shared Memory Communication over RDMA) is a representative example. Such approaches typically extend Socket protocol support inside the kernel, exchange RDMA metadata over a TCP connection, and automatically fall back to TCP when RDMA is unavailable, thereby ensuring transparent compatibility for applications. Their advantage lies in a complete compatibility path and tight integration with the existing system-call interface. However, they still find it difficult to avoid the extra copying and synchronization overhead on the kernel-space path, and their strong coupling with kernel implementation also limits deployment flexibility and scalability under high concurrency.

In contrast, user-space solutions usually intercept Socket library calls by means such as LD_PRELOAD [31,32,33,34] and directly access hardware through the RDMA Verbs interface, thereby bypassing the kernel protocol stack. These approaches are more flexible to deploy and have greater potential to reduce the overhead caused by kernel context switching [4,5,35,36]. NVIDIA’s Libvma [6] is one of the earlier representative works. Libvma constructs lightweight TCP/IP processing logic based on LWIP in user space and combines it with the underlying RDMA transport to provide a relatively high degree of Socket-compatible semantics for upper-layer applications. Its limitation is that software protocol processing introduces noticeable CPU overhead. More importantly, Libvma relies on raw-Socket-type QPs at the underlying communication layer and is therefore fundamentally oriented toward the InfiniBand hardware ecosystem; as a result, it does not support the more widely deployed RoCEv2 networks, which greatly restricts its interoperability and deployment scope [37].

In addition to Libvma, other studies have explored user-space RDMA compatibility and abstraction from different perspectives. For example, SocksDirect [4], vSocket [10], and TSoR [8] respectively target multi-threaded connection management, virtualized environments, and containerized scenarios, while RSocket [11] attempts to provide an interface closer to the traditional Socket abstraction, thereby lowering the barrier for applications to use RDMA Verbs. However, RSocket still exhibits clear limitations in receive-buffer organization: its data transfer relies on a shared receive buffer and sliding-window semantics, and if the receiver does not process data in time, the sender may continue writing and overwrite data that has not yet been consumed by the receiver, thereby introducing a receive-buffer overwrite risk. On the other hand, works such as RB^2^ [5] explore more efficient pointer synchronization and memory management from the perspective of distributed ring buffers and batching mechanisms. Although such studies do not directly provide a complete Socket compatibility layer, they offer important references for the design of high-performance user-space communication systems. Overall, although existing user-space approaches have made significant progress in high-performance communication, many of them still rely on relatively heavy software protocol processing, centralized resource coordination, or specific hardware and runtime environments, leaving room for further optimization in resource-constrained and latency-sensitive edge-intelligence scenarios.

### 2.3. Research Gap and Positioning of This Work

In summary, existing “Socket over RDMA” studies have already provided multiple implementation paths for enabling traditional network applications to exploit the high-performance RDMA data plane, yet several issues remain insufficiently addressed. First, most existing solutions are primarily designed for resource-rich datacenter environments, where their design assumptions rely on relatively strong CPU, memory, and system software support. In contrast, in latency-critical edge-intelligence scenarios such as autonomous driving, teleoperation, and industrial edge analytics, node resources are more constrained, making the overhead introduced by centralized resource coordination, heavy software protocol processing, and complicated control paths much more pronounced [38,39,40]. Second, although many compatibility solutions preserve the Socket programming interface to a certain extent, their data planes still depend on relatively heavy software protocol processing, centralized monitoring components, or specific runtime environments, making it difficult to simultaneously achieve deployment transparency, runtime lightweightness, and system scalability. Third, existing work still shows deficiencies in event notification, preservation of I/O multiplexing semantics, and the mapping from low-level asynchronous completion states to upper-layer Socket readiness semantics. As a result, many approaches can provide some degree of transmission acceleration, yet cannot fully support the transparent migration of existing applications [41,42,43].

Based on the above analysis, this paper focuses on a lightweight Socket–RoCE transparent compatibility mechanism for edge-intelligence scenarios. Different from existing work, this paper does not attempt to rebuild a complete software TCP/IP stack in user space. Instead, with preservation of standard Socket semantics as a prerequisite, it is designed around user-space interception, data transfer without conventional protocol-stack processing, decentralized resource management, and unified event handling, aiming to transparently accelerate existing Socket applications with low software overhead in resource-constrained environments. Specifically, the core issues addressed in this work include how to efficiently map stream-oriented Socket communication semantics onto a RoCE-based high-performance data plane without modifying applications, how to avoid bottlenecks caused by centralized monitoring and re-synchronization paths, and how to effectively convert low-level asynchronous completion events into application-visible synchronous I/O semantics. These issues together constitute the starting point for the subsequent system design and implementation presented in this paper.

## 3. Overall Architecture Design of TransBridge

The main purpose of TransBridge is to enable existing Socket-based applications to transparently benefit from the high-performance communication capability of RoCE without requiring any modification to their source code. To achieve this goal, TransBridge is designed to preserve standard Socket semantics while minimizing the software overhead introduced by conventional protocol-stack processing and runtime coordination.

**Figure 2:**
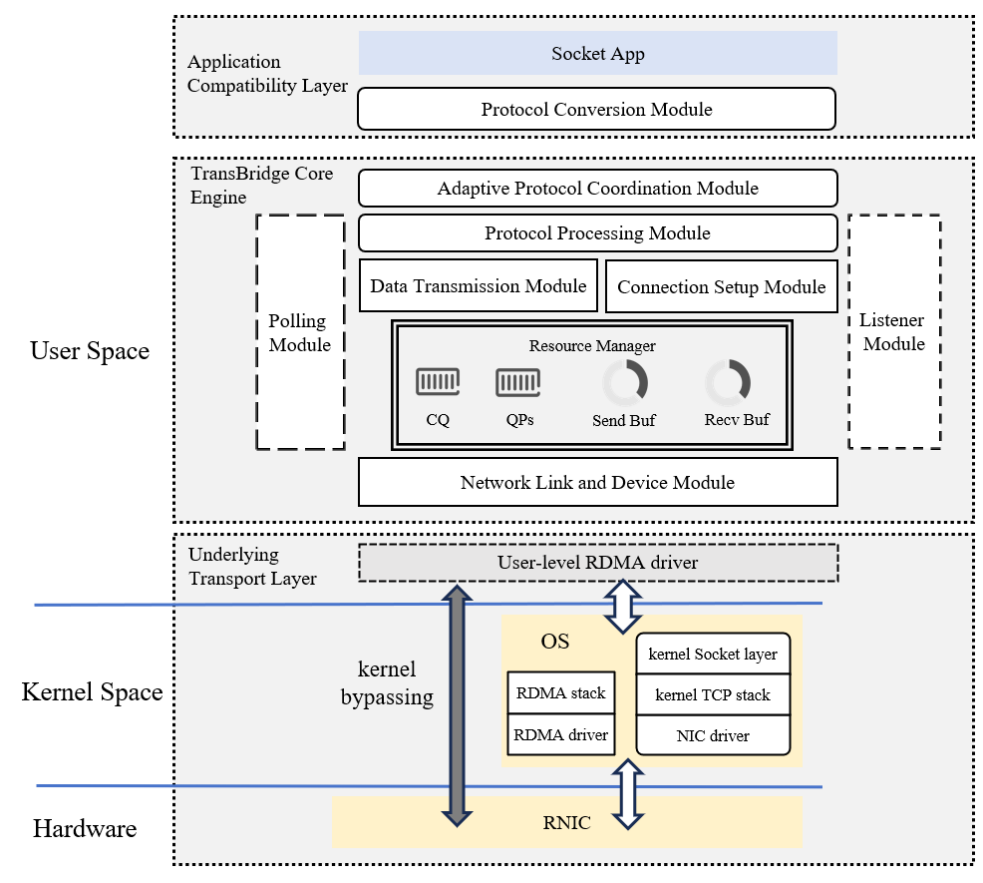
Architecture of TransBridge.

As shown in Figure 2, TransBridge is positioned between the application and the underlying network transport layer. It preserves the standard Socket programming interface at the upper layer while utilizing both the kernel TCP path and the RoCE high-performance data path at the lower layer. Applications still perform network programming through standard interfaces such as socket(), connect(), accept(), send(), and recv(), whereas TransBridge transparently intercepts these interface calls in user space and redirects eligible communication requests to its internal high-performance processing path without requiring any modification to application code. This interception mechanism is implemented through dynamic library loading based on LD_PRELOAD, which is simple to realize and does not require kernel modification. At the same time, the dynamic library can either be loaded by default when a process starts or be manually injected when an application is launched, providing high flexibility in both deployment and usage. In this manner, applications are not required to understand or directly use the RDMA Verbs programming model; instead, low-level logic such as queue pairs, memory registration, completion events, and resource management is encapsulated inside the middleware, thereby enabling existing applications to transparently benefit from RoCE acceleration while preserving their original programming interface and usage model.

From an overall organizational perspective, TransBridge does not simply replace TCP entirely with RoCE; instead, it adopts a dual-path architecture in which the control path and the data path are separated but work cooperatively. Specifically, the kernel TCP path mainly undertakes control-plane functions, including connection negotiation, metadata exchange, exception handling, and fallback support when necessary, while the RoCE path mainly carries actual payload transmission on the data plane. The reason for retaining the TCP control path is that connection establishment, exception awareness, and compatibility fallback in the standard Socket semantics are naturally better supported by the mature TCP mechanism, whereas once the connection relationship and the data-plane metadata have been prepared, subsequent payload transmission can be offloaded to RoCE. Through this division of responsibilities, the system forms an overall organization in which the control path preserves compatibility and robustness, while the data path pursues high performance and low overhead. This design not only avoids the complexity of reconstructing a complete software TCP/IP stack in user space, but also allows the system to retain the ability to fall back to the traditional path when acceleration conditions are not satisfied or when underlying resources are unavailable.

Around the above dual-path cooperative structure, TransBridge internally organizes several core functional modules, including connection management, resource management, data transmission, event handling, and exception fallback, and completes the mapping from Socket semantics to the RoCE data plane through the collaboration among these modules. The connection management module maintains the correspondence between application-level connection objects and underlying transport resources, and coordinates the establishment and state maintenance of both the control path and the data path. The resource management module organizes queue pairs, memory regions, send buffers, receive buffers, and the necessary context information associated with each connection. The data transmission module undertakes the send()/recv() semantic requests issued by applications and maps them to high-performance RoCE-oriented transmission operations. The event handling module uniformly receives low-level completion events, control-channel events, and necessary internal state changes, and converts these asynchronous events into application-visible Socket readable/writable states. The exception fallback module switches the corresponding communication process back to the kernel TCP path when acceleration conditions are not satisfied, link abnormalities occur, or underlying resources become unavailable, thereby maintaining connection availability and overall system robustness.

## 4. Design

This section presents the detailed design of TransBridge from four aspects: transparent connection establishment, decentralized resource management, protocol-stack-less data transmission, and unified event handling. Together, these components enable TransBridge to preserve standard Socket semantics while providing a lightweight and high-performance RoCE-based communication path.

### 4.1. Transparent Connection Establishment and Metadata Exchange

**Figure 3:**
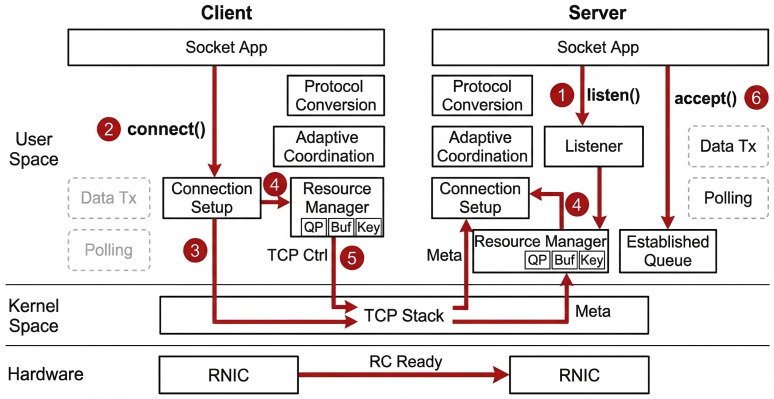
Transparent connection establishment and metadata exchange in TransBridge.

For an RC (Reliable Connection)-based RoCE data path, both communication endpoints must obtain the peer’s Queue Pair Number (QPN), receive buffer address, Remote Key (RKey), buffer size, and connection identifier before data transmission begins. Since the standard TCP/Socket connection establishment process does not include such low-level resource information, TransBridge introduces an additional metadata exchange phase beneath the standard connect()/accept() semantics. As shown in Figure 3, transparent connection establishment in TransBridge is completed through the following sequence: server listening, client control-connection initiation, local resource initialization, metadata exchange, data-path binding, and application-level retrieval of an already established connection.

The complete procedure consists of the following six steps:

**Step 1: The server enters the listening state.** The server application first calls listen() to enter the listening state so that incoming control-connection requests from clients can be continuously received.

**Step 2: The client issues a standard connect() call.** After the client application calls connect(), TransBridge initiates a control-connection request to the server along the standard TCP path.

**Step 3: The server listener thread receives the connection request.** After receiving the incoming control-connection request from the client, the server listener thread establishes the corresponding TCP control connection in the background and advances the subsequent connection setup procedure.

**Step 4: Both sides initialize local RoCE resources.** After the TCP control connection is established, the client and the server each create a local queue pair for the current connection, register a receive buffer, and generate the local QPN, receive buffer address, RKey, buffer size, and connection identifier.

**Step 5: Both sides exchange metadata and complete connection binding.** After local resource initialization, the client and the server exchange their QPN, receive buffer address, RKey, buffer size, and connection identifier through the established TCP control connection. After receiving the peer metadata, both sides bind the current Socket connection object to the RoCE data path, and the server inserts the fully established connection into the established-connection queue.

**Step 6: The server returns an already established connection through accept().** When the server application calls accept(), TransBridge directly retrieves and returns a connection object that has already completed control-connection establishment, metadata exchange, and data-path binding from the established-connection queue. On the client side, connect() returns after the corresponding connection setup is completed.

In this way, TransBridge completes server listening, control-connection establishment, RoCE resource preparation, metadata exchange, connection binding, and established-connection return under the standard Socket connection establishment flow.

### 4.2. Decentralized Resource Management and Lock-Free Buffer Update Mechanism

**Figure 4:**
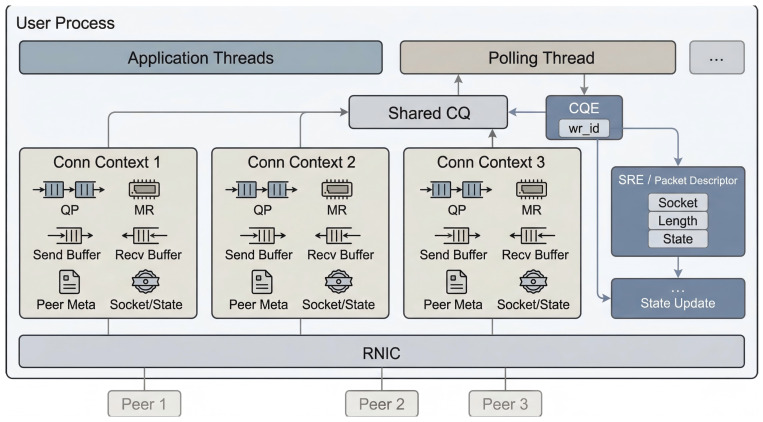
Decentralized resource management and connection-private context in TransBridge.

After transparent connection establishment is completed, TransBridge further organizes the resources and states required for subsequent data transmission on a per-connection basis. Instead of relying on a centralized monitoring process or global coordination tables, TransBridge adopts a decentralized resource management scheme: each connection maintains an independent connection context, within which the corresponding queue pair, registered memory region, send buffer, receive buffer, and peer metadata are all bound together; meanwhile, completion events are received through a shared completion queue at the process level. As illustrated in Figure 4, data-plane resources are organized as connection-private entities, whereas the event reception path is shared within the process, thereby avoiding the extra synchronization and serialization overhead introduced by centralized resource coordination.

Under this organization, TransBridge uses the wr_id field provided by RoCE Work Requests to establish a direct mapping from hardware completion events to software operation contexts. Specifically, wr_id is not an automatically assigned sequential identifier inside the protocol stack; rather, it is a 64-bit user-defined field attached to each Work Request at submission time. Once the Work Request is completed, the same wr_id is returned by the hardware in the corresponding Completion Queue Entry (CQE). As shown in Figure 4, TransBridge does not use wr_id merely as a conventional numeric identifier, but directly stores the address of the software request entity associated with the current transmission in wr_id. Since wr_id in RoCE is 64 bits wide, it can directly carry a pointer value. Therefore, when constructing a send or receive request, the system directly assigns the address of the corresponding SRE (Send/Receive Request Entry) or packet descriptor object to wr_id. This object further records the Socket connection to which the current transfer belongs, the transfer length, and other necessary state information.

When the background polling thread retrieves a CQE from the shared completion queue, it first reads the returned wr_id and then reconstructs the corresponding SRE or packet descriptor through a simple type cast, without requiring any secondary lookup through global hash tables, index tables, or identifier mapping tables. Furthermore, the polling thread can directly obtain the connection identifier and the completed byte count from this object, and then update the send-buffer or receive-buffer state of the corresponding connection accordingly. In the case of send completion, the system can accumulate the acknowledged length into the send state of the corresponding connection and, during subsequent merging, advance head, thereby releasing the corresponding buffer space; in the case of receive or control notifications, the same object can likewise be used to directly locate the associated connection and perform the corresponding state update.

**Figure 5:**
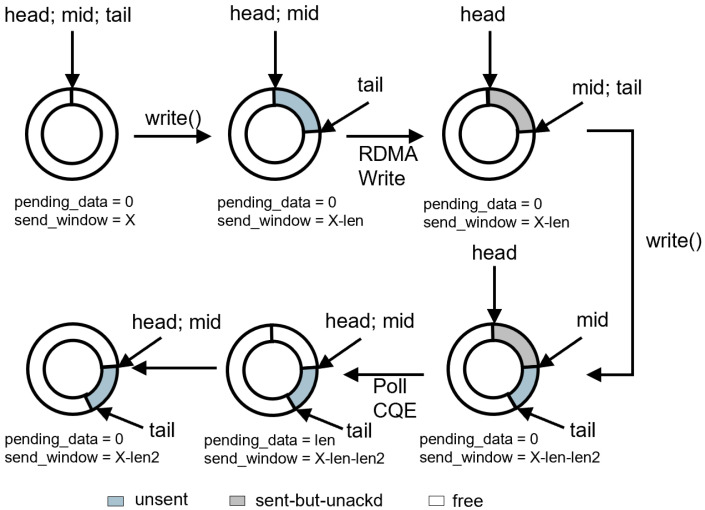
Lock-free ring buffer and deferred update mechanism in TransBridge.

On top of this, TransBridge moves the semantics of traditional Socket send/receive buffers into user space and maintains a pair of independent lock-free ring buffers for each connection. As shown in Figure 5, the send buffer adopts a three-pointer state organization: head denotes the starting position of the sent-but-unacknowledged region, mid denotes the starting position of the unsent region, and tail denotes the starting position of the free region. Accordingly, the ring buffer at any moment is divided into three parts: the sent-but-unacknowledged region, the unsent region, and the free region. When the application thread writes data, tail is advanced; when the background sending path submits a RoCE Write, mid is advanced; and after send completion, the corresponding data is not immediately reclaimed into the free region, but is released only after a subsequent acknowledgment merge advances head. The receive buffer, by contrast, adopts a read/write-separated organization: the lower-level write path advances the readable range, while the application-side read path advances the consumption position and releases receive-buffer space.

The reason for adopting this lock-free organization is that the buffer state of a single connection may be accessed concurrently by application threads, the sending thread, and the polling thread. If every send completion or every application read were to immediately update all shared pointers and window metadata, frequent lock contention and atomic synchronization overhead would arise under high-throughput workloads. To address this issue, TransBridge adopts a deferred update strategy: the fast path performs only lightweight state advancement or atomic accumulation, while the full merge of shared window state and statistical information is postponed until the accumulated amount reaches a threshold, free space becomes insufficient, or a critical operation occurs. In this way, state correctness is preserved while synchronization overhead is reduced.

The core of this deferred update strategy lies in delayed window maintenance. As illustrated in Figure 5, once the polling thread retrieves a CQE, it immediately advances the head pointer, because application threads do not access head when writing data and therefore no lock contention exists on this pointer. However, the polling thread does not immediately update the send-window size. Instead, it atomically accumulates the newly acknowledged bytes into a pending-confirmation counter. This is because application threads—that is, the threads issuing Socket APIs such as send() and write(), potentially multiple threads in the same process—frequently access the send window when transmitting data. Under high-throughput workloads, if the polling thread were to continuously update the send window as well, severe contention would arise between the polling thread and the application threads, resulting in substantial bandwidth waste. Therefore, the actual send-window update is deferred until a subsequent application thread detects that the remaining window space is insufficient and then actively merges the pending acknowledged bytes to refresh the visible send-window state. In this way, contention between the application threads and the polling thread is greatly reduced, with almost no noticeable impact on overall system performance.

### 4.3. Protocol-Stack-Less Data Transmission Mechanism

On top of the decentralized resource organization and lock-free buffer abstraction described above, TransBridge further constructs a protocol-stack-less data transmission mechanism that preserves TCP byte-stream semantics. The fundamental motivation for this design is that the conventional kernel TCP data plane still follows a full software path of “application write—kernel buffering—layered protocol-stack processing—peer-side reception—application read,” which incurs extra overhead from system calls, user-kernel transitions, buffer copying, and layered software processing. Even if the protocol stack is moved into user space, the transmission path still needs to undertake complete TCP/IP header processing, reliability maintenance, and flow-control logic in software, so the data-plane processing cost does not fundamentally disappear [44,45]. Table 1 compares the protocol-processing differences among different transmission schemes on the data plane.

**Figure 6:**
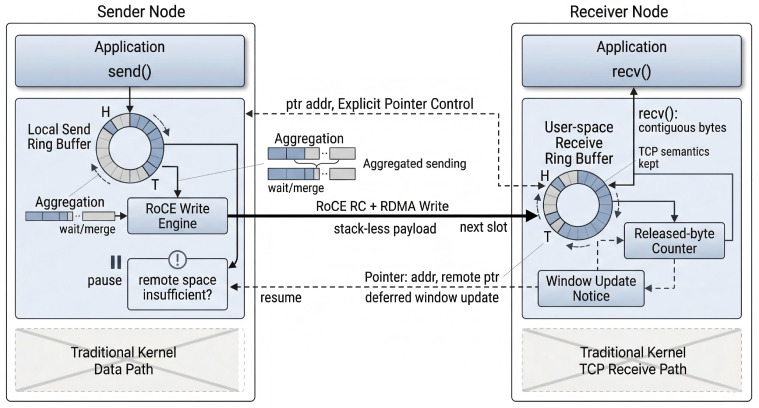
Protocol-stack-less data transmission, aggregated sending, window update, and transmission resumption in TransBridge.

Unlike conventional kernel protocol stacks and user-space software protocol-stack approaches, TransBridge does not attempt to rebuild a complete software TCP/IP stack in user space. Instead, while preserving standard Socket programming semantics, it migrates the actual payload traffic onto a RoCE RC-based data plane, where ordered submission, reliable delivery, and low-level retransmission are handled by hardware, while user space retains only the lightweight logic necessary to preserve TCP byte-stream semantics, such as buffer organization, aggregated sending, and receiver-window feedback. As illustrated in Figure 6, when the application calls send(), data is first written into the local send buffer; when the remote receive window allows further writes, the system extracts pending data from the local send buffer and issues RoCE Write operations to place the data directly into the peer’s user-space receive buffer; when the application calls recv(), the system reads contiguous bytes from the local receive buffer and returns them to the application. In this way, the traditional TCP data path of “application write—kernel buffer—protocol-stack processing—peer reception—application read” is reconstructed into a user-space closed loop of “local buffering—remote direct write—local read,” while the application still observes standard Socket byte-stream semantics.

Within this data path, TransBridge adopts a sender-side aggregation mechanism instead of directly mapping each application-level send() call to an independent RDMA operation. In the system, data written by the application first enters the local send buffer, and the background sending path determines when to actually trigger transmission based jointly on the accumulated pending-data volume in the buffer and an aggregation timer. In the current implementation, the sender uses a low-water threshold of 1 KB and a high-water threshold of 8 KB. Meanwhile, the system maintains a base aggregation waiting time $T_{base}$ for each connection according to the measured normal transmission latency between the two communicating nodes. When the accumulated pending data remains below the low-water threshold, the system uses $T_{base}$ as the current aggregation waiting time; when the accumulated pending data reaches the high-water threshold, transmission is triggered immediately; when the accumulated pending data lies between the low-water and high-water thresholds, the system proportionally shortens the aggregation waiting time according to the current buffered size, so that the closer the pending data is to the high-water threshold, the shorter the remaining waiting time becomes. Accordingly, the current aggregation waiting time is defined as

$$T_{wait}\left(x\right)=\left\{\begin{array}{cc} T_{base}, & x\leq 1\;\mathrm{KB},\\ T_{base}\cdot \frac{8\;\mathrm{KB}-x}{8\;\mathrm{KB}-1\;\mathrm{KB}}, & 1\;\mathrm{KB}<x<8\;\mathrm{KB},\\ 0, & x\geq 8\;\mathrm{KB}, \end{array}\right.$$

where *x* denotes the amount of contiguous pending data currently accumulated in the send buffer. In this way, the sending path no longer relies on a fixed waiting time, but dynamically determines the transmission timing according to both the inter-node link latency and the local pending-data volume.

Based on the above triggering mechanism, TransBridge preferentially merges adjacent contiguous data segments in the send buffer into larger write intervals and then completes transmission using fewer RoCE Write requests, thereby reducing the overhead caused by fine-grained Work Request submission, CQ processing, and hardware queue scheduling. If the aggregation timer expires before the accumulated pending data reaches the high-water threshold, the system still sends the currently accumulated data rather than waiting indefinitely. Meanwhile, aggregated transmission is always constrained by the remote receive window: when the remote window is insufficient to support a new write, the system suspends further submission instead of continuing to transmit blindly; once a later window update arrives, the sender rechecks the current pending-data volume against the available window space and resumes transmission according to the same dynamic waiting rule. In this way, TransBridge jointly incorporates the local pending-data volume, the normal inter-node transmission latency, and the remote receiving capability into the send-side decision process, thereby balancing throughput efficiency and transmission latency.

Correspondingly, the receiver adopts an organization in which data first arrives in the user-space receive buffer and is then read by the application. RoCE Write operations issued by the sender no longer traverse the traditional kernel TCP receive path on the peer side, but instead place the payload directly into the user-space receive buffer associated with the corresponding connection at the receiver. Since this buffer has already been registered during connection establishment, and since its starting address, RKey, and capacity have been exchanged with the sender through the control channel, the sender can write data to the correct receive offset according to the locally maintained remote receive-window view. When the application invokes recv(), TransBridge directly reads the contiguous bytes that have already arrived but have not yet been consumed from the local receive buffer, and copies them into the destination memory provided by the application. From the application’s perspective, this still appears as standard Socket byte-stream reading semantics, without exposing the underlying RDMA write boundaries.

Each time recv() consumes data, the receiver releases the corresponding receive-buffer space, but the sender cannot automatically learn the newly available writable range. Therefore, TransBridge maintains an accumulated released-byte counter at the receiver and adopts a threshold-based deferred window notification mechanism. Instead of sending a window update immediately after every recv() return, the system first accumulates the newly released space in local state, and only when the accumulated released amount reaches a threshold does it send a window update back to the sender. This feedback is implemented through a data-plane notification carrying immediate data, where the flag bits in the immediate-data field distinguish ordinary data from window notifications, and the remaining bits carry the effective length released this time. After receiving the notification, the sender updates its locally maintained remote receive-window view and determines whether the data previously suspended due to insufficient window space can now resume transmission.

Although TransBridge constructs a protocol-stack-less data path in user space, it does not give up reliable transmission semantics. First, the underlying data plane uses the RoCE RC transport type, so ordered request submission, transmission reliability, and low-level retransmission are guaranteed by hardware, eliminating the need to reconstruct a complete software-reliable transport protocol in user space. Second, during runtime, the sender still needs to maintain its local sending state, the remote receive-window view, and the pending state to be merged from completion events and window notifications, so that the sending range, progress advancement, and buffer-space reclamation remain consistent. When the sender detects that the remaining remote receive-window space is insufficient, the system still allows the application to write data into the local send buffer, but it does not immediately submit additional RoCE Write requests. For blocking Sockets, if local free space is also exhausted, the send call waits at that point; for non-blocking Sockets, the corresponding standard Socket status is returned. Once a later window update arrives, the sender rechecks the relationship between local pending data and available window space and resumes transmission according to the aggregation rule; when a send completion event returns, the system then merges the completed length in a unified manner and releases the corresponding buffer space. As a result, window updates, send resumption, and progress advancement form a closed loop, ensuring reliable, ordered, and recoverable byte-stream transmission semantics even without a full software protocol stack.

### 4.4. Unified Event Handling and Preservation of Socket Semantics

To preserve the standard Socket programming interface while coordinating the RoCE-based data plane with the TCP-based control plane, TransBridge introduces a unified event handling mechanism in user space. Completion queue events, control-channel state transitions, receiver-window update notifications, and internal timer expirations are all incorporated into a single event processing framework. Once an event is captured, the background thread identifies the associated connection according to the event type and its context information, and then updates the corresponding sending state, receiving state, window state, or connection state. In this way, asynchronous state transitions that would otherwise be scattered across different modules are consolidated into a single connection progress path, avoiding the synchronization overhead and semantic fragmentation caused by maintaining multiple independent event paths.

On top of this unified framework, the polling frequency of the background thread is dynamically adjusted to balance event response latency and CPU overhead. Specifically, within each detection window Δ, the system records the number of CQ events, control events, window notification events, and timer events, and defines the aggregated event load as

$$L_{k}=N_{k}^{\left(cq\right)}+w_{c}N_{k}^{\left(ctrl\right)}+w_{w}N_{k}^{\left(win\right)}+w_{t}N_{k}^{\left(timer\right)},$$

from which the aggregated event rate in the current window is obtained as

$$\lambda_{k}=\frac{L_{k}}{\Delta }.$$

To prevent transient workload fluctuations from causing frequent oscillations in the polling frequency, TransBridge further smooths the event rate using an exponentially weighted moving average:

$${\hat{\lambda }}_{k}=\left(1-\alpha \right){\hat{\lambda }}_{k-1}+\alpha \lambda_{k}.$$

Based on the smoothed event rate, TransBridge allows the polling thread to switch among three operating modes: busy polling, interval polling, and idle waiting. When ${\hat{\lambda }}_{k}$ remains above a high threshold for consecutive detection windows, the thread enters busy polling mode and continuously checks for new events with zero sleep time. Under moderate workloads, the thread operates in interval polling mode, where the polling interval is dynamically determined by the recent event activity and can be expressed as

$$T_{poll,k}=\mathrm{clamp}\left(\frac{\kappa {\hat{g}}_{k}}{1+\eta \rho_{k}},\;T_{\min},\;T_{\max}\right),$$

where ${\hat{g}}_{k}$ denotes the smoothed estimate of the average event arrival gap, $\rho_{k}$ denotes the current pending-work pressure factor, and $T_{\min}$ and $T_{\max}$ denote the minimum and maximum polling intervals, respectively. When no event arrives for a sufficiently long time and no pending task remains, the thread enters idle waiting mode and is reactivated only when a new event is delivered. To avoid frequent mode transitions when the workload fluctuates around a threshold, hysteresis thresholds and consecutive-window confirmation are applied between different modes.

After unified event progression is completed, TransBridge further maps the underlying state changes to application-visible Socket semantics. When consecutive readable bytes are available in the receive buffer, the corresponding connection is marked as readable. When the send buffer has available space and the remote receive window allows further transmission, the connection is marked as writable. When connection establishment completes, blocking connect() or accept() returns successfully, whereas in non-blocking mode the application is notified through readiness events. Likewise, when underlying failures, timeouts, or connection loss is detected, the system translates them into corresponding Socket error states. As a result, applications do not need to be aware of low-level CQ events, window feedback, or internal timer mechanisms, and can continue to operate transparently with standard Socket calls and event semantics.

## 5. Evaluation

This chapter presents the experimental evaluation of TransBridge. The evaluation covers three aspects: latency for small-message communication, throughput under continuous transmission, and end-to-end communication latency in a practical middleware scenario. Accordingly, sockperf (version 3.7) (https://github.com/Mellanox/sockperf (accessed on 15 April 2026)) is used for latency micro-benchmarks, iperf3 (version 3.16) (https://github.com/esnet/iperf (accessed on 15 April 2026)) is used for throughput tests, and application-level experiments are conducted in a DDS middleware scenario. Through these three parts, we examine the behavior of TransBridge in small-message low-latency communication, continuous data transfer, and existing Socket-based applications.

Three types of tests are conducted in this chapter. First, sockperf is used to evaluate system latency. Second, iperf3 is used to measure system throughput. Third, DDS is used for application-level evaluation, where both throughput and latency are measured under different data sizes to examine the impact of TransBridge on upper-layer application communication performance.

DDS is a commonly used publish/subscribe middleware in distributed systems and is suitable for representing middleware communication scenarios in existing network applications. In the application-level evaluation, Fast DDS version 2.8.2 (eProsima, Madrid, Spain) is used, with Fast CDR version 1.1.1 (eProsima, Madrid, Spain) as the serialization component and foonathan Memory (commit 2b2bdb2) (https://github.com/foonathan/memory (accessed on 15 April 2026)) as the memory allocation component. DDS can operate over different underlying transports, such as TCP and UDP. Since the goal of this work is to preserve standard Socket/TCP semantics and provide transparent acceleration for existing Socket-based applications, the DDS experiments in this chapter are conducted in the TCP-based setting. This part of the evaluation is used to further examine the impact of TransBridge on the communication path of existing distributed middleware.

The compared schemes include Linux TCP (Linux kernel TCP/IP stack, version 5.4.18), SMC-R (Shared Memory Communications over RDMA, Linux kernel version 5.4.18), and Libvma (version 9.8.51, NVIDIA Networking, Yokneam, Israel). Linux TCP is used as the conventional implementation of the standard Socket communication path; SMC-R represents kernel-space schemes, while Libvma represents user-space schemes. In part of the result analysis, native RDMA Write is also included as a reference to indicate the performance level that can be achieved by the underlying hardware without preserving full Socket semantics, and to analyze the gap between TransBridge and this reference result.

All experiments are conducted on the same 100 Gbps RoCE platform (RoCE v2 standard). The testbed consists of two nodes, each equipped with a Phytium D2000 eight-core processor (Phytium Technology Co., Ltd., Tianjin, China) and 32 GB of memory, running Kylin V10 SP1 (Kylinsoft Corporation, Tianjin, China) with Linux kernel version 5.4.18. The network uses Mellanox ConnectX-5 RoCE NICs (Mellanox Technologies, Ltd., Sunnyvale, CA, USA) with firmware version 16.32.1010, and the two nodes are interconnected through an Arista 7060CX-32S 100G switch (Arista Networks, Inc., Santa Clara, CA, USA). This board-level platform is used instead of a general-purpose high-end server in order to align the evaluation setting with the edge-node scenario considered in this paper. The problem addressed in this work concerns how to map standard Socket applications to a RoCE-based high-performance communication path with low software overhead under constrained CPU and memory resources. Therefore, the experimental platform is also chosen to match this setting.

### 5.1. Latency Micro-Benchmark

This subsection uses sockperf to measure the round-trip latency of different transmission schemes. The experiments are conducted on the 100 Gbps RoCE platform described above. The compared schemes include Linux TCP, SMC-R, Libvma, TransBridge, and native RDMA Write as a reference result. The message sizes range from 16 B to 16 KB.

**Figure 7:**
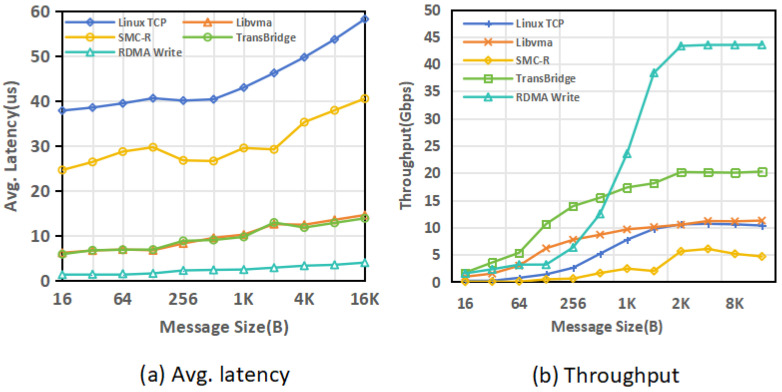
Latency and Througuput.

Figure 7a shows the average round-trip latency of different schemes under different message sizes. As the message size increases, the latency of all schemes increases, but the growth rates differ. TransBridge maintains lower average latency throughout the tested range, remaining below Linux TCP and SMC-R and overall outperforming Libvma. For 16 B messages, the average round-trip latency of TransBridge is 5.926 μs.

Table 2 further reports the latency distribution for two representative message sizes, 16 B and 2 KB. The results show that TransBridge not only achieves lower average latency, but also maintains better high-percentile latency. For 16 B messages, its 99% percentile latency is 6.519 μs, lower than 8.938 μs for Libvma, 25.893 μs for SMC-R, and 40.793 μs for Linux TCP. At the 99.99% percentile, TransBridge reaches 21.609 μs, which is also lower than the other three schemes. For 2 KB messages, the 99% and 99.99% percentile latencies of TransBridge are 13.177 μs and 32.065 μs, respectively, both lower than those of Linux TCP, SMC-R, and Libvma. These results indicate that TransBridge provides lower tail latency for representative message sizes.

This result is related to the data-path design of TransBridge. Linux TCP still relies on full software protocol stack processing. SMC-R still retains a kernel-space Socket–RDMA path. Libvma operates in user space, but it still undertakes software protocol processing. In contrast, TransBridge maps payload transmission onto a RoCE RC-based data plane and reduces data-plane software processing overhead while preserving Socket byte-stream semantics, thereby achieving lower latency.

It should also be noted that native RDMA Write still achieves lower latency than TransBridge. This gap is mainly caused by two factors. First, the current implementation of TransBridge still retains one user-space memory copy. Second, applications using native RDMA Write are typically developed directly on top of the RDMA programming interface and do not go through the middleware conversion from standard Socket semantics to the RDMA data plane, whereas TransBridge must complete this mapping while preserving Socket compatibility. Therefore, a certain performance gap between TransBridge and native RDMA Write is expected.

### 5.2. Throughput Benchmark

Figure 7b shows the throughput of different schemes under different message sizes. As the message size increases, the throughput of all schemes increases, but the growth rates differ. TransBridge achieves higher throughput in medium and large message scenarios. For 16 KB messages, the throughput of TransBridge reaches 20.254 Gbps, which is higher than 10.341 Gbps for Linux TCP, 11.252 Gbps for Libvma, and 4.656 Gbps for SMC-R. This result indicates that TransBridge can make more effective use of the RoCE data plane in continuous transmission scenarios.

This result is related to the sending-path design of TransBridge. First, TransBridge adopts an aggregation mechanism at the sender side, which merges adjacent continuous data segments in the send buffer and then submits larger RoCE Write requests, thereby reducing the overhead of fine-grained Work Request submission, CQ processing, and hardware queue scheduling. Second, TransBridge adopts a deferred window maintenance mechanism, which avoids frequent contention on the send window between application threads and the polling thread in high-throughput scenarios, thereby reducing synchronization overhead. In addition, TransBridge maps payload transmission onto a RoCE RC-based data plane and avoids full software TCP/IP protocol-stack processing in the data plane, which helps achieve higher throughput for larger messages. We further note that, in our early implementation, a lock-based version of TransBridge showed throughput behavior similar to that of Libvma. The substantially improved throughput of the current version is therefore closely related to the adoption of the lock-free and deferred-update design, which helps reduce contention on the critical path.

### 5.3. Application-Level Evaluation with DDS

For the throughput test, the benchmark tool provided by Fast DDS is used. Since this benchmark mainly reports application-level throughput, its results are directly used as the throughput reference in the DDS scenario. For the latency test, we further implement a custom test program based on DataWriter and DataReader to measure the average round-trip latency at the application layer. Table 3 summarizes the application-level throughput and average round-trip latency of different schemes in the DDS scenario.

As shown in Table 3, in the DDS throughput test, the results of the non-accelerated scheme, Libvma, SMC-R, and TransBridge are 98.840 Mbps, 101.014 Mbps, 80.413 Mbps, and 188.561 Mbps, respectively. Compared with the non-accelerated scheme, TransBridge achieves higher application-level throughput in this scenario. It also outperforms Libvma and SMC-R in terms of throughput. For the average round-trip latency, the non-accelerated scheme reaches 633.594 μs, while Libvma and SMC-R achieve about 200 μs and 400 μs, respectively. In contrast, TransBridge achieves about 150 μs. These results show that TransBridge can also reduce application-level communication latency in the DDS scenario.

It should be noted that the DDS results here are not equivalent to the raw throughput and latency of the underlying transmission path itself. On the one hand, Fast DDS carries its own protocol headers during communication. On the other hand, the test process also includes additional overheads such as serialization, deserialization, and internal middleware processing. Therefore, the results in Table 3 should be regarded as application-level reference results rather than bare performance metrics of the underlying data transmission path.

Nevertheless, these experiments still show that TransBridge can support the normal operation of the DDS system while preserving standard Socket/TCP semantics, and that the acceleration achieved at the Socket layer can be further reflected in the communication performance of upper-layer distributed middleware. For this work, the main point of this result is not to provide the absolute performance upper bound of the DDS system, but to show that TransBridge can not only transparently adapt existing Socket-based applications, but also bring measurable performance improvement in an actual middleware scenario.

## 6. Conclusions

This paper addresses the practical problem that existing Socket-based applications on resource-constrained edge nodes cannot directly leverage the high-performance data plane of RoCE, and designs and implements TransBridge, a user-space transparent communication middleware. While preserving the standard Socket/TCP programming model, TransBridge establishes a transparent bridge between Socket semantics and the RoCE data plane through a user-space interception mechanism, allowing existing applications to use the accelerated communication path without modification. To achieve this goal, the system is designed from three aspects: system architecture, data transmission, and runtime management. First, a dual-path architecture is adopted, in which the TCP path is responsible for connection negotiation, metadata exchange, and exception fallback, while the RoCE path carries the actual payload transmission. Second, a protocol-stack-less data transmission mechanism for TCP byte-stream semantics is constructed. Through intermediate buffering, aggregated sending, and receive-window feedback, high-performance transmission based on RoCE RC is achieved without rebuilding a full software TCP/IP protocol stack. Third, a decentralized resource management and lock-free buffer update mechanism is proposed. Combined with unified event handling, it maps low-level asynchronous completion states to application-visible standard Socket semantics, thereby reducing runtime overhead caused by lock contention and centralized coordination.

Experimental results show that, while preserving compatibility with the standard Socket API, TransBridge achieves better latency and throughput performance than representative existing schemes. In the micro-benchmark tests, TransBridge achieves an average round-trip latency of 5.926 μs for 16 B messages and a throughput of 20.254 Gbps for 16 KB messages. In the DDS middleware scenario, TransBridge can also support normal system operation and propagate the acceleration effect of the Socket layer to upper-layer applications, achieving an application-level throughput of 188 Mbps and an average round-trip latency of about 150 μs, both better than the non-accelerated scheme as well as Libvma and SMC-R. These results indicate that TransBridge can not only reduce communication overhead in micro-benchmark scenarios, but also provide performance gains in practical distributed middleware environments.

There is still room for further improvement in the current work. To preserve full compatibility with the standard Socket API, the current implementation of TransBridge still retains one user-space memory copy. In addition, further testing and optimization are still needed to improve adaptability under real edge-network conditions, such as bandwidth fluctuations, complex topologies, and more diverse application workloads. Moreover, the current design and evaluation mainly focus on TCP scenarios. Since UDP differs significantly from TCP in connection establishment, data exchange patterns, and state management logic, future work is needed to further study adaptation mechanisms for UDP, so as to extend TransBridge to support different Socket communication semantics. Future work will therefore focus on reducing the remaining copy operations in the data path, improving system adaptability under complex network conditions, extending validation to more real edge-application scenarios, and further supporting other transport semantics such as UDP, in order to enhance the generality and performance of TransBridge in practical deployments.

## Figures and Tables

**Table 1:** Differences in data-plane protocol processing among different transmission schemes.

Scheme	Transmission Form	Header Processing	Description
Kernel TCP/IP	Transmission through the kernel TCP/IP stack	Full TCP/IP header processing	High software overhead on the data plane.
Libvma	Transmission through IB raw Socket	Still requires full software header processing	Kernel bypassed, but software overhead remains.
TransBridge	RoCE RC + RDMA Write	No full software TCP/IP data-plane header processing	Data transmission is mainly offloaded to hardware.

**Table 2:** Latency statistics of different transmission schemes under 16 B and 2 KB message sizes.

**(a) 16 B**					
**Pct.**	**Socket**	**Libvma**	**TB**	**SMC**	**RDMA**
max	304.236	170.004	74.217	161.536	1.425
99.999	223.609	96.982	53.746	109.529	1.411
99.99	125.274	28.334	21.609	57.347	1.399
99.9	53.564	15.917	10.333	34.038	1.388
99	40.793	8.938	6.519	25.893	1.380
90	36.813	8.376	6.110	23.660	1.373
75	35.500	8.188	5.981	22.636	1.366
50	34.334	8.000	5.845	21.946	1.360
25	33.647	7.854	5.789	21.535	1.354
min	27.875	7.250	5.286	18.504	1.350
**(b) 2 KB**					
**Pct.**	**Socket**	**Libvma**	**TB**	**SMC**	**RDMA**
max	1678.121	153.899	184.319	387.096	7.305
99.999	325.798	117.545	87.979	156.931	5.903
99.99	150.503	58.335	32.065	86.131	4.765
99.9	69.730	35.938	20.854	45.015	3.713
99	53.272	24.521	13.177	30.719	2.925
90	46.522	18.813	11.413	26.736	2.911
75	43.168	18.355	10.942	25.689	2.896
50	42.230	17.980	10.819	25.372	2.884
25	41.293	17.667	10.680	24.933	2.873
min	36.584	15.959	9.861	23.076	2.865

**Table 3:** Application -level throughput and average round-trip latency of different schemes in the DDS scenario.

Scheme	Throughput (Mbps)	Avg. RTT (μs)
Linux TCP	98.840	633.594
Libvma	101.014	201.487
SMC-R	80.413	424.111
TransBridge	188.561	159.234

## Data Availability

The source code supporting this study is publicly available at https://github.com/zccccc1212/TransBri (accessed on 15 April 2026).

## Abbreviations

The following abbreviations are used in this manuscript:

TCP	Transmission Control Protocol
RoCE	RDMA over Converged Ethernet
RDMA	Remote Direct Memory Access
API	Application Programming Interface
DDS	Data Distribution Service
SMC-R	Shared Memory Communication over RDMA
QP	Queue Pair
MR	Memory Region
CQ	Completion Queue
CQE	Completion Queue Entry
WR	Work Request
SGE	Scatter/Gather Entry
QPN	Queue Pair Number
RKey	Remote Key
LKey	Local Key
RC	Reliable Connection
UD	Unreliable Datagram
NIC	Network Interface Card
SRE	Send Request Entry
UDP	User Datagram Protocol

## References

[1] Zhao Y., Wang W., Li Y., Meixner C., Tornatore M., Zhang J. (2019). Edge computing and networking: A survey on infrastructures and applications. IEEE Access.

[2] Shi W., Cao J., Zhang Q., Li Y., Xu L. (2016). Edge computing: Vision and challenges. IEEE Internet Things J..

[3] InfiniBand Trade Association (2014). InfiniBand Trade Association Releases Updated Specification for Remote Direct Memory Access over Converged Ethernet (RoCE). https://www.prweb.com/releases/infiniband_trade_association_releases_updated_specification_for_remote_direct_memory_access_over_converged_ethernet_roce_/prweb12172312.htm.

[4] Li B., Cui T., Wang Z., Bai W., Zhang L. (2019). Socksdirect: Datacenter sockets can be fast and compatible. Proceedings of the ACM Special Interest Group on Data Communication.

[5] Sun H., Tan Y., Wu Y., Zhu J., Huang Q., Yao X., Zhang G. (2024). RB 2: Narrow the Gap between RDMA Abstraction and Performance via a Middle Layer. Proceedings of the IEEE INFOCOM 2024—IEEE Conference on Computer Communications.

[6] Mellanox libvma: Messaging Accelerator (VMA). https://github.com/mellanox/libvma.

[7] Fox M., Kassimis C., Stevens J. (2015). IBM’s Shared Memory Communications over RDMA (SMC-R) Protocol. RFC 7609. https://www.rfc-editor.org/info/rfc7609.

[8] Sun Y., Qu Q., Zhao C., Krishnamurthy A., Chang H., Xiong Y. (2023). TSoR: TCP Socket over RDMA Container Network for Cloud Native Computing. arXiv.

[9] Ren Z., Fan M., Wang Z., Zhang J., Zeng C., Huang Z., Hong C., Chen K. Accelerating Secure Collaborative Machine Learning with Protocol-Aware RDMA. Proceedings of the 33rd USENIX Security Symposium (USENIX Security 24).

[10] Wang D., Fu B., Lu G., Tan K., Hua B. vSocket: Virtual socket interface for RDMA in public clouds. Proceedings of the 15th ACM SIGPLAN/SIGOPS International Conference on Virtual Execution Environments.

[11] (2022). Linux Man Page. Rsocket—Rdma Socket API. https://linux.die.net/man/7/rsocket.

[12] Pinkerton J., Deleganes E., Krause M. (2003). Sockets Direct Protocol (SDP) for iWARP over TCP (v1.0). https://www.rdmaconsortium.org/home/draft-pinkerton-iwarp-sdp-v1.0.pdf.

[13] Kim D., Yu T., Liu H., Zhu Y., Padhye J., Raindel S., Guo C., Sekar V., Seshan S. FreeFlow: Software-based virtual RDMA networking for containerized clouds. Proceedings of the 16th USENIX Symposium on Networked Systems Design and Implementation (NSDI 19).

[14] Pinkerton J. Software RDMA Over Converged Ethernet. https://github.com/SoftRoCE.

[15] Skiadopoulos A., Xie Z., Zhao M., Cai Q., Agarwal S., Adelmann J., Ahern D., Contavalli C., Goldflam M., Mayatskikh V. High-throughput and flexible host networking for accelerated computing. Proceedings of the 18th USENIX Symposium on Operating Systems Design and Implementation (OSDI 24).

[16] Wei X., Lu F., Chen R., Chen H. KRCORE: A microsecond-scale RDMA control plane for elastic computing. Proceedings of the 2022 USENIX Annual Technical Conference (USENIX ATC 22).

[17] Reda W., Canini M., Kostić D., Peter S. RDMA is Turing complete, we just did not know it yet!. Proceedings of the 19th USENIX Symposium on Networked Systems Design and Implementation (NSDI 22).

[18] Wang Z., Ma T., Kong L., Wen Z., Li J., Song Z., Lu Y., Chen G., Cao W. Zero overhead monitoring for cloud-native infrastructure using RDMA. Proceedings of the 2022 USENIX Annual Technical Conference (USENIX ATC 22).

[19] Geng L., Wang H., Meng J., Fan D., Ben-Romdhane S., Pichumani H., Phegade V., Zhang X. (2024). RR-Compound: RDMA-Fused gRPC for Low Latency, High Throughput, and Easy Interface. IEEE Trans. Parallel Distrib. Syst..

[20] Tan L., Su W., Liu Y., Gao X., Zhang W. (2021). DCQUIC: Flexible and reliable software-defined data center transport. Proceedings of the IEEE INFOCOM 2021—IEEE Conference on Computer Communications Workshops (INFOCOM WKSHPS).

[21] Abaza H., Habishyashi A., Roy D., Bastoni A., Hammadeh Z.A.H., Fan S., Saidi S., Tverdyshev S. (2023). RDMA-Based Deterministic Communication Architecture for Autonomous Driving. 2023 IEEE 29th International Conference on Embedded and Real-Time Computing Systems and Applications (RTCSA).

[22] Ichikawa J., Yamaguchi T., Mochida Y., Masutani H., Tonomura Y., Takahashi H. (2024). Remote Robot Control with Haptic Feedback Enabled by Low-latency Transport and Precision Bilateral Control Technologies. NTT Tech. Rev..

[23] Li X., Shu R., Xiong Y., Ren F. (2025). Software-based Live Migration for RDMA. Proceedings of the ACM SIGCOMM 2025 Conference.

[24] Peng D., Liu C., Palit T., Vahldiek-Oberwagner A., Vij M., Fonseca P. Pegasus: Transparent and Unified Kernel-Bypass Networking for Fast Local and Remote Communication. Proceedings of the Twentieth European Conference on Computer Systems.

[25] Zhang H., Zhang H., Zhang L., Wu Y. (2021). FastUDP: A highly scalable user-level UDP framework in multi-core systems for fast packet I/O. J. Supercomput..

[26] Adam D. (2001). Design and Implementation of the lwIP TCP/IP Stack. https://www.nongnu.org/lwip/.

[27] Jeong E., Wood S., Jamshed M., Jeong H., Ihm S., Han D., Park K. mTCP: A highly scalable user-level TCP stack for multicore systems. Proceedings of the 11th USENIX Symposium on Networked Systems Design and Implementation (NSDI 14).

[28] Huang Y., Geng J., Lin D., Wang B., Li J., Ling R., Li D. Los: A high performance and compatible user-level network operating system. Proceedings of the First Asia-Pacific Workshop on Networking.

[29] Dragojević A., Narayanan D., Castro M., Hodson O. FaRM: Fast remote memory. Proceedings of the 11th USENIX Symposium on Networked Systems Design and Implementation (NSDI 14).

[30] Li Y., Miao R., Liu H., Zhuang Y., Feng F., Tang L., Cao Z., Zhang M., Kelly F., Alizadeh M. HPCC: High precision congestion control. Proceedings of the ACM Special Interest Group on Data Communication.

[31] Kaufmann A., Stamler T., Peter S., Sharma N., Krishnamurthy A., Anderson T. TAS: TCP acceleration as an OS service. Proceedings of the Fourteenth EuroSys Conference 2019.

[32] Soares L., Stumm M. FlexSC: Flexible system call scheduling with Exception-Less system calls. Proceedings of the 9th USENIX Symposium on Operating Systems Design and Implementation (OSDI 10).

[33] Wang Z., Luo L., Ning Q., Zeng C., Li W., Wan X., Xie P., Feng T., Cheng K., Geng X. SRNIC: A scalable architecture for RDMA NICs. Proceedings of the 20th USENIX Symposium on Networked Systems Design and Implementation (NSDI 23).

[34] Li T., Shi H., Lu X. HatRPC: Hint-accelerated thrift RPC over RDMA. Proceedings of the International Conference for High Performance Computing, Networking, Storage and Analysis.

[35] Lu Y., Chen G., Li B., Tan K., Xiong Y., Cheng P., Zhang J., Chen E., Moscibroda T. Multi-Path transport for RDMA in datacenters. Proceedings of the 15th USENIX symposium on networked systems design and implementation (NSDI 18).

[36] Burke M., Dharanipragada S., Joyner S., Szekeres A., Nelson J., Zhang I., Ports D. PRISM: Rethinking the RDMA interface for distributed systems. Proceedings of the ACM SIGOPS 28th Symposium on Operating Systems Principles.

[37] Kalia A., Kaminsky M., Andersen D. Design guidelines for high performance RDMA systems. Proceedings of the 2016 USENIX annual technical conference (USENIX ATC 16).

[38] Planeta M., Bierbaum J., Roitzsch M., Härtig H. (2023). CoRD: Converged RDMA Dataplane for High-Performance Clouds. arXiv.

[39] Sahoo S., Chava S. (2024). Scatter Protocol: An Incentivized and Trustless Protocol for Decentralized Federated Learning. Proceedings of the 2024 IEEE International Conference on Blockchain (Blockchain).

[40] Goldenberg D., Kagan M., Ravid R., Tsirkin M. (2005). Zero copy sockets direct protocol over infiniband-preliminary implementation and performance analysis. Proceedings of the 13th Symposium on High Performance Interconnects (HOTI’05).

[41] Handley M., Raiciu C., Agache A., Voinescu A., Moore A., Antichi G., Wójcik M. Re-architecting datacenter networks and stacks for low latency and high performance. Proceedings of the Conference of the ACM Special Interest Group on Data Communication.

[42] DPDK Project (2018). Data Plane Development Kit. https://www.dpdk.org/.

[43] Yi B., Xia J., Chen L., Chen K. Towards zero copy dataflows using RDMA. Proceedings of the SIGCOMM Posters and Demos.

[44] Rosa L., Foschini L., Corradi A. (2024). Empowering cloud computing with network acceleration: A survey. IEEE Commun. Surv. Tutor..

[45] Addanki V., Bai W., Schmid S., Apostolaki M. Reverie: Low pass Filter-Based switch buffer sharing for datacenters with RDMA and TCP traffic. Proceedings of the 21st USENIX Symposium on Networked Systems Design and Implementation (NSDI 24).

